# Warfarin Dosing and Time Required to Reach Therapeutic International Normalized Ratio in Patients with Hypercoagulable Conditions

**DOI:** 10.4274/tjh.2015.0271

**Published:** 2016-12-01

**Authors:** Pushpinderdeep Kahlon, Shahzaib Nabi, Adeel Arshad, Absia Jabbar, Ali Haythem

**Affiliations:** 1 Wayne State University, Henry Ford Health System, Clinic of Internal Medicine, Detroit, USA; 2 Weill Cornell University, Hamad Medical Corporation, Clinic of Internal Medicine, Doha, Qatar; 3 Nishtar Hospital, University of Health Science, Multan, Pakistan; 4 Wayne State University, Henry Ford Health System, Clinic of Hematology-Oncology, Detroit, USA

**Keywords:** International normalized ratio, Warfarin, Hypercoagulable conditions, venous thromboembolism

## Abstract

**Objective:**

The purpose of this study was to analyze the difference in duration of anticoagulation and dose of warfarin required to reach a therapeutic international normalized ratio [(INR) of 2 to 3] in patients with hypercoagulable conditions as compared to controls. To our knowledge, this study is the first in the literature to delineate such a difference.

**Materials and Methods:**

A retrospective chart review was performed in a tertiary care hospital. The total study population was 622. Cases (n=125) were patients with a diagnosis of a hypercoagulable syndrome who developed venous thromboembolism. Controls (n=497) were patients with a diagnosis of venous thromboembolism in the absence of a hypercoagulable syndrome and were matched for age, sex, and race.

**Results:**

The total dose of warfarin required to reach therapeutic INR in cases was higher (50.7±17.6 mg) as compared to controls (41.2±17.7 mg). The total number of days required to reach therapeutic INR in cases was 8.9±3.5 days as compared to controls (6.8±2.9 days). Both of these differences were statistically significant (p<0.001).

**Conclusion:**

Patients with hypercoagulable conditions require approximately 10 mg of additional total warfarin dose and also require, on average, 2 extra days to reach therapeutic INR as compared to controls.

## Abstract (tr)

**Amaç:**

Bu çalışmanın amacı kontrollerle karşılaştırıldığında hiperkoagülabilite durumları olan hastalarda terapötik uluslararası düzeltme oranında (INR) 2 ile 3 aralığına ulaşmak için gerekli warfarin doz ve antikoagülan süresindeki farklılığı analiz etmektir. Bildiğimiz kadarıyla; bu farklılığı tarifleyen literatürdeki ilk çalışmadır.

**Gereç ve Yöntemler:**

Retrospektif dosya taraması 3. basamak hastanede yapıldı. Toplam çalışmaya alınan hasta sayısı 622 idi. Venöz tromboembolizmi olan bu hastalardan 125’inin hiperkoagülabilite sendromu olup yaş, cins ve etnik kökeni aynı 497 kontrol hastasında hiperkoagülabilite sendromu yoktu.

**Bulgular:**

Hastalarda terapötik INR’ye ulaşmak için gerekli total warfarin dozu (50,7±17,6 mg) kontrollerin dozu (41,2±17,7 mg) ile karşılaştırıldığında yüksekti. Terapötik INR’ye ulaşmak için gerekli total gün sayısı hastalarda 8,9±3,5 gün olup kontrollerde 6,8±2,9 gün idi. Her iki karşılaştırmada da istatistiksel farklılık anlamlı bulundu (p<0,001).

**Sonuç:**

Hiperkoagülabilite durumları olan hastalarda terapötik INR’ye ulaşmak için kontrollere göre yaklaşık 10 mg ek total warfarin dozu ve ortalama 2 ek gün gereklidir.

## INTRODUCTION

It has been well documented that both acquired and hypercoagulable conditions play an important role in thrombophilia development. Studies suggest that important genetic factors that have notable significance include factor V Leiden mutation, prothrombin gene mutation, deficiency of protein S or protein C, antithrombin III deficiency, and hyperhomocysteinemia. Acquired hypercoagulability factors include non-modifiable factors, such as age and antiphospholipid antibodies, and modifiable factors, such as pregnancy, oral contraceptive and hormone replacement therapy, recent travel, and obesity, as well other factors such as malignancy, recent surgery, trauma, and prolonged immobility [1].

Once a patient develops venous thromboembolism (VTE), the main mode of treatment has been warfarin, with recent advent of newer medications such as rivaroxaban [2]. Warfarin still remains one of the most commonly used medications for VTE in the United States. Previously there have been a few studies that have investigated warfarin dosing in specific hypercoagulable conditions, such as antiphospholipid antibodies and high- versus low-intensity warfarin efficacy in recurrent deep vein thrombosis (DVT) prevention [3]. However, it is not known if a difference exists in the total dose and time of warfarin therapy necessary to reach a therapeutic international normalized ratio (INR) in patients with hypercoagulable conditions. The goal of this study was to determine the difference in the time and dose of warfarin required to reach therapeutic INR (i.e. INR of 2 to 3) in patients with hypercoagulable conditions as compared to controls.

## MATERIALS AND METHODS

The study was approved by our institutional review board. A retrospective chart review was performed for patients seen in our tertiary care facility from January 2002 to December 2012. The inclusion criteria for cases were patients with hypercoagulable conditions, which included patients with factor V Leiden mutation, prothrombin gene mutation, protein S or protein C deficiency, antithrombin III deficiency, dysfibrinogenemia, and antiphospholipid antibodies who developed unprovoked VTE (DVT, pulmonary embolism, or both). The diagnostic tests used were venous duplex for DVT and computed tomography angiogram or ventilation/perfusion lung scan for pulmonary embolism. Controls were age-, sex-, and race-matched patients who developed VTE but did not have a hypercoagulable syndrome. Confounding factors were assessed in both cases and controls and included end-stage renal disease, malignancies, recent surgery (within 1 month of development of VTE), and oral contraceptive use. Therapeutic INR was defined as an INR of 2-3 on 2 consecutive blood draws separated by a 24-h duration. All subjects received an initial 5-mg loading dose of warfarin and all of them received heparin at the time of diagnosis of VTE (bridging therapy). The total dose of warfarin required to reach a therapeutic INR and the number of days required to reach a therapeutic INR were analyzed.

Statistical analysis with a primary aim of comparing cases to controls was performed. Data were described using standard descriptive statistics, i.e. counts, percentages, means, and standard deviations. Crude (unadjusted) odds ratios were obtained from univariate logistic regression models. All variables with a univariate p-value of <0.2 were placed in a multivariable logistic regression and stepwise selection with stay criteria of p≤0.05 were used to arrive at a final model. Statistical significance was set at p<0.05 and all analyses were performed using SAS 9.4 (SAS Institute Inc., Cary, NC, USA).

## RESULTS

A total of 622 patients were analyzed in this study. Of these, 125 were cases and 497 were controls. The mean age at the time of diagnosis of VTE in both cases and controls was 53 years. In all, 58% of the patients were female. The male to female ratios for both the cases and controls were roughly the same. The most common race was Caucasian (59%), followed by African American (32%); other races constituted 9% of the total study population. Among the patient population, 39% developed a DVT, 42% developed a pulmonary embolism, and 18% developed both a DVT and a pulmonary embolism.

The total number of days required to reach therapeutic INR was 8.9±3.5 days in cases, whereas in controls it was 6.8±2.9 days. The difference was found to be statistically significant (p<0.001). The total dose of warfarin required to reach therapeutic INR was 50.7±17.6 mg in cases as compared to 41.2±17.7 mg in controls. The difference remained statistically significant after multivariate regression analysis (p<0.001).

A multivariable model was built starting with all variables with a univariate p-value of <0.2. Stepwise selection was then used to arrive at the final model given in Table 1. We found that every 1-day increase in the number of days to therapeutic INR was associated with 19% increased odds of being a case, and every 1-unit increase in warfarin dose to therapeutic INR was associated with 1% increased odds of being a case.

## DISCUSSION

For the last 60 years, warfarin has been the mainstay of management of thromboembolism in a variety of both hereditary and acquired conditions [4]. Even with its narrow therapeutic index, meticulous monitoring, dire adverse effects, and interactions with an array of foods, drugs, and herbs, warfarin is still the most widely used oral anticoagulant in North America with over 25 million prescriptions in the United States in 2010 [4,5].

Warfarin acts by interfering with the enzyme vitamin K epoxide reductase, which modulates the gamma carboxylation of procoagulant factors II, VII, IX, and X and anticoagulant proteins C, S, and Z [6]. Because of the latter action, warfarin has the potential of exerting a transient procoagulant effect early in therapy. To counter that, heparin ‘bridging’ is recommended for a minimum of 5 days and until the INR is 2.0 or above for at least 24 h [7]. As the antithrombotic effect of warfarin necessitates the inhibition of factor II, which has a very long half-life (60-72 h) as compared to other factors (6-24 h), it takes approximately 6 days for warfarin to exert its full efficacy even though the earliest changes in INR can be seen after 24 to 36 h [8,9,10,11]. The average number of days to achieve therapeutic INR after starting warfarin is reported to be 5-6 days [12].

Selection of an appropriate dose for warfarin initiation is challenging and controversial because of interpersonal variability in its pharmacokinetic and pharmacodynamic parameters. Kovacs et al. found that patients who were initiated with 10 mg of warfarin achieved therapeutic INR 1.4 days earlier than those who received 5 mg [13]. One study concluded that initiation with 5 mg of warfarin was associated with 5.6 days of bridging with low-molecular-weight heparin [14]. The American College of Chest Physicians recommends initiation with 10 mg in patients healthy enough to be treated as outpatients, with dose modifications done as per the INR after 2 days [7]. From a practical point of view, adjusting the warfarin dose to achieve and maintain therapeutic INR is a challenging task that we face regularly during our day-to-day clinical encounters. A myriad of factors lead to this commonly observed interpatient variation in the warfarin dose requirement and number of days required to achieve the therapeutic INR. Our study compared these 2 variables in patients with and without hypercoagulable conditions. We found that patients with hypercoagulable conditions on average require higher doses and more days to achieve the target INR as compared to those without any hypercoagulable conditions. To our knowledge, this study is one of the first in the literature to delineate such a difference.

As described earlier, other factors might also affect the variables under study, which could have been potential confounders in our study. The elderly and females require a smaller weekly dose of warfarin than their counterparts. Even though there are no convincing data, it is generally preferred that the elderly be started on a low-dose warfarin regimen because of the exaggeration of anticoagulation response in this age group [15]. One of the strongest and statistically significant patient-specific factors that can influence the warfarin dose requirement is the concomitant use of drugs that affect cytochrome P450 (17.2 mg additional dosage of warfarin per week) [16]. From antibiotics to anticonvulsants, ginger to ginseng, and spinach to spices, a tiring list of drugs, herbs, and foods is reported to interact with warfarin by multiple mechanisms, which can involve its absorption, bioavailability, metabolism, and excretion. Recent surgery was also assessed as a variable in this study. It should be noted that surgeries are generally considered to be transiently hypercoagulable states. Surgeries involving lower extremities (such as hip/knee replacement) carry the highest risk of VTE and should be managed carefully in patients with hypercoagulable states.

Even though not recommended for general testing, genetic mutations can lead to variations in the dosage requirement of warfarin among different patients, which ultimately affects the number of days required to achieve the therapeutic INR. Polymorphism in the VKORC1 gene, which codes for the target enzyme for warfarin, results in 2 haplotypes: A, which makes the patient sensitive to smaller doses, and B, which necessitates administration of higher doses to achieve and maintain the same range of INR. The Asp36Tyr missense mutation in VKORC1, found in 15% of the Ethiopian population in one study, was strongly associated with a warfarin requirement of >70 mg/week. On the other hand, CYP2C9 (and less commonly CYP1A1, CYPCA1, and CYP3A4), which metabolizes the more potent enantiomer of the warfarin molecule, has been found to have 2 relatively common variant forms with reduced activity (CYP2CP*2 and CYP2C9*3). Patients with these variants have less rapid clearance of warfarin, thus requiring lower dosage administrations [17]. In one study, VKORC1 was significantly associated with the time required to achieve the first therapeutic INR while CYP2C9 predicted the time to reach an INR above 4, which predisposes the patient to hemorrhagic complications [18,19].

Gene polymorphisms are found to be more common in African Americans than Asians and Caucasians, which affects the number of days and the dose needed to achieve the first target INR. Other patient-specific factors that can affect the variables under study include body mass index/body surface area (especially height), poor compliance, comorbid conditions, and true warfarin resistance, which is a quite rare occurrence (0.01%) [19].

The major limitation of this study is that it was a single-center, retrospective study and the results might not be applicable to the general population. Moreover, our ‘cases’ group was relatively small, likely secondary to the rarity of the above-mentioned hypercoagulable conditions. However, to compensate for this relatively small sample size, we used a large ‘control’ group to increase the power of the study. Every effort was made during data collection to avoid bias as much as possible.

## CONCLUSION

In summary, this study lays the foundation of a novel idea of comparing warfarin dosage and the time required to achieve therapeutic INR in patients with and without known hypercoagulability conditions. The likely mechanism of the observed difference is inherent thrombogenic potential in hypercoagulable states with more natural resistance towards anticoagulation. With a few confounders playing a role, this proposition needs further consolidation with large-scale trials that might help us in predicting the initial dose to start with in patients with and without a procoagulant condition. The observed effect can, in another way, be studied retrospectively to understand the difference in the pathophysiology of the thromboembolism in these 2 populations, which may explain the etiological aspects of the results noticed.

## Acknowledgment

We would like to acknowledge the great efforts of our exceptionally hard-working librarian, Stephanie Stebens, who helped us in the final editing of this manuscript. Her suggestions played a huge role in finalizing this manuscript.

## Ethics

Ethics Committee Approval: The study was approved by the IRB/Ethics Committee; Informed Consent: Was not needed as this was a retrospective chart review.

## Figures and Tables

**Table 1:**
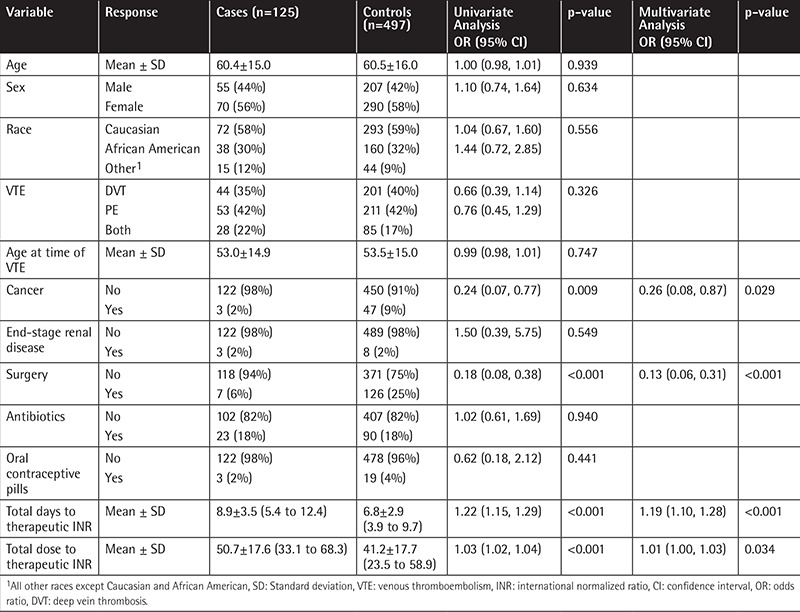
Patient characteristics along with univariate and multivariate analysis.
